# The Charter of the Texas State Medical Association

**Published:** 1889-07

**Authors:** 


### CHARTER T. S. M. A.

Pursuant to a resolution introduced by Dr. T. J. Bennett, at the late meeting of the State Association, and unanimously adopted, the President, Dr. R. M. Swearingen, and the Secretary, Dr. F. E. Daniel, applied, July 1st, to the Secretary of State for a Charter, and the same was immediately granted. The following is a copy of the

### ARTICLES OF INCORPORATION OF THE TEXAS STATE MEDICAL ASSOCIATION.

"*Know all men by these presents*, That we, R. M. Swearingen, F. E. Daniel and T. J. Bennett, all citizens of Travis county, Texas, and J. Larendon, a citizen of Harris county, Texas, and our associates, desiring to form ourselves into a body corporate and politic under the general laws of the State of Texas governing the creation of private corporations, hereby adopt and subscribe the following

CHARTER :

I. NAME.—The name of this corporation shall be, The Texas State Medical Association.

II. PURPOSES.—The purposes for which this corporation is created are:

1. To organize the regular medical profession of the State in the most efficient manner possible.

2. To encourage a high standard of professional qualification and ethics.

3. To promote professional brotherhood.

4. To labor for the advancement of State Medicine, i. e., of public hygiene; of medical education; of medical jurisprudence, and public institutions for the sick and infirm.

III. TERM OF EXISTENCE.—This corporation shall exist for the full period of fifty years.

IV. DOMICILE.—The principal office of this Association shall be at Austin, Travis county, Texas, or wherever the Secretary of said Association may reside.

V. TRUSTEES.—The business of the Association shall be managed by a board of nine trustees, and

J. F. Y. Paine, M. D., Galveston, Texas;
Sam R. Burroughs, M. D., Raymond, Texas;
O. Eastland, M. D., Wichita Falls, Texas;
W. L. York, M. D., Decatur, Texas;
E. L. Thompson, M. D., Dallas, Texas;
A. G. Clopton, M. D., Jefferson, Texas;
G. W. Kerr, M. D., Waelder, Texas;
*———— M. D.;
*———— M. D.

are hereby declared to be the trustees for the first year.

VI. CAPITAL STOCK.—This Association has no capital stock; the estimated value of its assets is fifty dollars.

Witness our hands this the 20th day of July, A. D. 1889.

(Signed) R. M. SWEARINGEN, M. D.,
F. E. DANIEL, M. D.,
T. J. BENNETT, M. D.,
J. LARENDON, M. D.

*To be appointed.

